# Association of p16 expression with prognosis varies across ovarian carcinoma histotypes: an Ovarian Tumor Tissue Analysis consortium study

**DOI:** 10.1002/cjp2.109

**Published:** 2018-09-21

**Authors:** Peter F Rambau, Robert A Vierkant, Maria P Intermaggio, Linda E Kelemen, Marc T Goodman, Esther Herpel, Paul D Pharoah, Stefan Kommoss, Mercedes Jimenez‐Linan, Beth Y Karlan, Aleksandra Gentry‐Maharaj, Usha Menon, Susanna Hernando Polo, Francisco J Candido dos Reis, Jennifer Anne Doherty, Simon A Gayther, Raghwa Sharma, Melissa C Larson, Paul R Harnett, Emma Hatfield, Jurandyr M de Andrade, Gregg S Nelson, Helen Steed, Joellen M Schildkraut, Micheal E Carney, Estrid Høgdall, Alice S Whittemore, Martin Widschwendter, Catherine J Kennedy, Frances Wang, Qin Wang, Chen Wang, Sebastian M Armasu, Frances Daley, Penny Coulson, Micheal E Jones, Micheal S Anglesio, Christine Chow, Anna de Fazio, Montserrat García‐Closas, Sara Y Brucker, Cezary Cybulski, Holly R Harris, Andreas D Hartkopf, Tomasz Huzarski, Allan Jensen, Jan Lubiński, Oleg Oszurek, Javier Benitez, Fady Mina, Annette Staebler, Florin Andrei Taran, Jana Pasternak, Aline Talhouk, Mary Anne Rossing, Joy Hendley, AOCS Group, Robert P Edwards, Sian Fereday, Francesmary Modugno, Roberta B Ness, Weiva Sieh, Mona A El‐Bahrawy, Stacey J Winham, Jenny Lester, Susanne K Kjaer, Jacek Gronwald, Peter Sinn, Peter A Fasching, Jenny Chang‐Claude, Kirsten B Moysich, David D Bowtell, Brenda Y Hernandez, Hugh Luk, Sabine Behrens, Mitul Shah, Audrey Jung, Prafull Ghatage, Jennifer Alsop, Kathryn Alsop, Jesús García‐Donas, Pamela J Thompson, Anthony J Swerdlow, Chloe Karpinskyj, Alicia Cazorla‐Jiménez, María J García, Susha Deen, Lynne R Wilkens, José Palacios, Andrew Berchuck, Jennifer M Koziak, James D Brenton, Linda S Cook, Ellen L Goode, David G Huntsman, Susan J Ramus, Martin Köbel

**Affiliations:** ^1^ Department of Pathology and Laboratory Medicine University of Calgary, Foothills Medical Center Calgary AB Canada; ^2^ Pathology Department Catholic University of Health and Allied Sciences‐Bugando Mwanza Tanzania; ^3^ Department of Health Sciences Research, Division of Biomedical Statistics and Informatics Mayo Clinic Rochester MN USA; ^4^ School of Women's and Children's Health Faculty of Medicine, University of NSW Sydney Sydney NSW Australia; ^5^ Department of Public Health Sciences Medical University of South Carolina Charleston SC USA; ^6^ Samuel Oschin Comprehensive Cancer Institute, Cancer Prevention and Genetics Program, Cedars‐Sinai Medical Center Los Angeles CA USA; ^7^ National Center for Tumor Diseases, University of Heidelberg Heidelberg Germany; ^8^ Centre for Cancer Genetic Epidemiology, Department of Oncology University of Cambridge Cambridge UK; ^9^ Centre for Cancer Genetic Epidemiology, Department of Public Health and Primary Care University of Cambridge Cambridge UK; ^10^ Department of Women's Health Tübingen University Hospital Tübingen Germany; ^11^ Department of Histopathology Addenbrookes Hospital Cambridge UK; ^12^ Women's Cancer Program at the Samuel Oschin Comprehensive Cancer Institute, Cedars‐Sinai Medical Center Los Angeles CA USA; ^13^ Gynaecological Cancer Research Centre, Women's Cancer, Institute for Women's Health, University College London London UK; ^14^ Medical Oncology Service Hospital Universitario Funcación Alcorcón Alcorcón Spain; ^15^ Department of Gynecology and Obstetrics, Ribeirão Preto Medical School University of São Paulo Ribeirão Preto Brazil; ^16^ Department of Population Health Sciences Huntsman Cancer Institute, University of Utah Salt Lake City UT USA; ^17^ Department of Preventive Medicine, Keck School of Medicine University of Southern California Los Angeles CA USA; ^18^ Center for Cancer Prevention and Translational Genomics Samuel Oschin Comprehensive Cancer Institute, Cedars‐Sinai Medical Center Los Angeles CA USA; ^19^ Department of Biomedical Sciences Cedars‐Sinai Medical Center Los Angeles CA USA; ^20^ Pathology West ICPMR Westmead Westmead Hospital, The University of Sydney Sydney NSW Australia; ^21^ University of Western Sydney at Westmead Hospital Westmead NSW Australia; ^22^ Centre for Cancer Research, The Westmead Institute for Medical Research, The University of Sydney Sydney NSW Australia; ^23^ The Crown Princess Mary Cancer Centre Westmead, Sydney‐West Cancer Network, Westmead Hospital Sydney NSW Australia; ^24^ Department of Oncology, Division of Gynecologic Oncology, Cumming School of Medicine University of Calgary Calgary AB Canada; ^25^ Department of Obstetrics and Gynecology, Division of Gynecologic Oncology Royal Alexandra Hospital Edmonton AB Canada; ^26^ Department of Public Health Sciences University of Virginia Charlottesville VA USA; ^27^ John A. Burns School of Medicine, Department of Obstetrics and Gynecology University of Hawaii Honolulu HI USA; ^28^ Department of Virus, Lifestyle and Genes Danish Cancer Society Research Center Copenhagen Denmark; ^29^ Molecular Unit, Department of Pathology Herlev Hospital, University of Copenhagen Copenhagen Denmark; ^30^ Department of Health Research and Policy – Epidemiology Stanford University School of Medicine Stanford CA USA; ^31^ Department of Biomedical Data Science Stanford University School of Medicine Stanford CA USA; ^32^ Department of Gynaecological Oncology Westmead Hospital Sydney NSW Australia; ^33^ Cancer Control and Population Sciences Duke Cancer Institute Durham NC USA; ^34^ Department of Community and Family Medicine Duke University Medical Center Durham NC USA; ^35^ Department of Health Sciences Research Mayo Clinic Rochester MN USA; ^36^ Division of Breast Cancer Research Institute of Cancer Research London UK; ^37^ Division of Bioscience Brunel University London UK; ^38^ Division of Genetics and Epidemiology Institute of Cancer Research London UK; ^39^ Department of Pathology and Laboratory Medicine University of British Columbia Vancouver BC Canada; ^40^ Genetic Pathology Evaluation Centre, Vancouver General Hospital and University of British Columbia Vancouver BC Canada; ^41^ Division of Cancer Epidemiology and Genetics National Cancer Institute Bethesda MD USA; ^42^ Department of Gynecology and Obstetrics University of Tübingen Tübingen Germany; ^43^ Department of Genetics and Pathology Pomeranian Medical University Szczecin Poland; ^44^ Program in Epidemiology, Division of Public Health Sciences Fred Hutchinson Cancer Research Center Seattle WA USA; ^45^ Department of Environmental Medicine, Division of Nutritional Epidemiology Karolinska Institutet Stockholm Sweden; ^46^ International Hereditary Cancer Center, Department of Genetics and Pathology Pomeranian Medical University Szczecin Poland; ^47^ Human Cancer Genetics Programme Spanish National Cancer Research Centre (CNIO) Madrid Spain; ^48^ Biomedical Network on Rare Diseases (CIBERER) Madrid Spain; ^49^ Institute of Pathology, Tübingen University Hospital Tübingen Germany; ^50^ British Columbia's Ovarian Cancer Research (OVCARE) Program Vancouver General Hospital, BC Cancer Agency and University of British Columbia Vancouver BC Canada; ^51^ Department of Epidemiology University of Washington Seattle WA USA; ^52^ Department of Research, Cancer Genomics and Genetics Peter MacCallum Cancer Center Melbourne VIC Australia; ^53^ Peter MacCallum Cancer Center Melbourne VIC Australia; ^54^ Department of Genetics and Computational Biology QIMR Berghofer Medical Research Institute Brisbane QLD Australia; ^55^ Ovarian Cancer Center of Excellence, Womens Cancer Research Program Magee‐Womens Research Institute and University of Pittsburgh Cancer Institute Pittsburgh PA USA; ^56^ Division of Gynecologic Oncology, Department of Obstetrics, Gynecology and Reproductive Sciences University of Pittsburgh School of Medicine Pittsburgh PA USA; ^57^ Womens Cancer Research Center, Magee‐Womens Research Institute and Hillman Cancer Center Pittsburgh PA USA; ^58^ University of Texas MD Anderson Cancer Center Houston TX USA; ^59^ Department of Genetics and Genomic Sciences, Department of Population Health Science and Policy, Icahn School of Medicine at Mount Sinai New York NY USA; ^60^ Department of Histopathology, Imperial College London Hammersmith Hospital London UK; ^61^ Department of Gynaecology, Rigshospitalet University of Copenhagen Copenhagen Denmark; ^62^ Department of Pathology Institute of Pathology, University Hospital Heidelberg Heidelberg Germany; ^63^ David Geffen School of Medicine, Department of Medicine Division of Hematology and Oncology University of California at Los Angeles Los Angeles CA USA; ^64^ Department of Gynecology and Obstetrics Comprehensive Cancer Center ER‐EMN, University Hospital Erlangen, Friedrich‐Alexander‐University Erlangen‐Nuremberg Erlangen Germany; ^65^ Division of Cancer Epidemiology German Cancer Research Center (DKFZ) Heidelberg Germany; ^66^ Cancer Epidemiology Group University Cancer Center Hamburg (UCCH), University Medical Center Hamburg‐Eppendorf Hamburg Germany; ^67^ Division of Cancer Prevention and Control Roswell Park Cancer Institute Buffalo NY USA; ^68^ Sir Peter MacCallum Department of Oncology The University of Melbourne Parkville VIC Australia; ^69^ Cancer Epidemiology Program University of Hawaii Cancer Center Honolulu HI USA; ^70^ Medical Oncology Service HM Hospitales – Centro Integral Oncológico HM Clara Campal Madrid Spain; ^71^ Division of Genetics and Epidemiology The Institute of Cancer Research London UK; ^72^ Division of Breast Cancer Research The Institute of Cancer Research London UK; ^73^ Pathology Department Fundación Jiménez Díaz‐Quiron Salud Madrid Spain; ^74^ Department of Histopathology Queen's Medical Centre, Nottingham University Hospitals NHS Trust Nottingham UK; ^75^ Pathology Department, IRYCIS, CIBERONC Universidad de Alcalá, Hospital Universitario Ramón y Cajal Madrid Spain; ^76^ Department of Obstetrics and Gynecology Duke University Medical Center Durham NC USA; ^77^ Alberta Health Services‐Cancer Care Calgary AB Canada; ^78^ Cancer Research UK Cambridge Institute, University of Cambridge Cambridge UK; ^79^ University of New Mexico Health Sciences Center, University of New Mexico Albuquerque NM USA; ^80^ Department of Cancer Epidemiology and Prevention Research Alberta Health Services Calgary AB Canada; ^81^ Department of Health Science Research, Division of Epidemiology Mayo Clinic Rochester MN USA; ^82^ Department of Molecular Oncology BC Cancer Agency Research Centre Vancouver BC Canada; ^83^ The Kinghorn Cancer Centre, Garvan Institute of Medical Research Sydney NSW Australia

**Keywords:** ovary, immunocytochemistry, RT‐QPCR

## Abstract

We aimed to validate the prognostic association of p16 expression in ovarian high‐grade serous carcinomas (HGSC) and to explore it in other ovarian carcinoma histotypes. p16 protein expression was assessed by clinical‐grade immunohistochemistry in 6525 ovarian carcinomas including 4334 HGSC using tissue microarrays from 24 studies participating in the Ovarian Tumor Tissue Analysis consortium. p16 expression patterns were interpreted as abnormal (either overexpression referred to as block expression or absence) or normal (heterogeneous). CDKN2A (which encodes p16) mRNA expression was also analyzed in a subset (*n* = 2280) mostly representing HGSC (*n* = 2010). Association of p16 expression with overall survival (OS) was determined within histotypes as was CDKN2A expression for HGSC only. p16 block expression was most frequent in HGSC (56%) but neither protein nor mRNA expression was associated with OS. However, relative to heterogeneous expression, block expression was associated with shorter OS in endometriosis‐associated carcinomas, clear cell [hazard ratio (HR): 2.02, 95% confidence (CI) 1.47–2.77, *p* < 0.001] and endometrioid (HR: 1.88, 95% CI 1.30–2.75, *p* = 0.004), while absence was associated with shorter OS in low‐grade serous carcinomas (HR: 2.95, 95% CI 1.61–5.38, *p* = 0.001). Absence was most frequent in mucinous carcinoma (50%), and was not associated with OS in this histotype. The prognostic value of p16 expression is histotype‐specific and pattern dependent. We provide definitive evidence against an association of p16 expression with survival in ovarian HGSC as previously suggested. Block expression of p16 in clear cell and endometrioid carcinoma should be further validated as a prognostic marker, and absence in low‐grade serous carcinoma justifies CDK4 inhibition.

## Introduction

*CDKN2A* (cyclin‐dependent kinase inhibitor 2A) is located on chromosome 9p21.3 and encodes two proteins, p16 and p14^ARF^, that have different reading frames 1. p14^ARF^ inhibits p53 function and p16 inhibits the CDK4/6 complex acting as a negative cell cycle regulator suppressing the transition from the Gap1 to DNA synthesis (G1/S) phase and arresting the cell cycle in the G1 phase 2. Normal cells express variable amounts of p16 protein that can be detected by immunohistochemistry (IHC) in both nuclear and cytoplasmic localizations (heterogeneous p16 expression pattern) 3. There are two abnormal p16 expression patterns: absent and overexpressed, the latter also referred to as block expression as recommended by the Lower Anogenital Squamous Terminology Standardization Project for HPV‐Associated Lesions (LAST) 4. In keeping with its role as a tumor suppressor, absence of p16 expression can occur due to various mechanisms including homozygous deletion, loss of function mutations, promoter hypermethylation and translational suppression 5. In ovarian carcinoma, homozygous deletion of *CDKN2A* has been detected in only 3% of high‐grade serous carcinomas (HGSC) 6, 15% of low‐grade serous carcinomas (LGSC) 7, and in 30% of mucinous carcinomas (MC) 8. In contrast, p16 block expression results from a variety of alterations in G1/S cell cycle transition as a compensatory effort to inhibit G1/S transition. p16 block expression is classically observed in human papillomavirus (HPV)‐associated uterine cervical neoplasms, in which viral proteins (E7) inactivate pRB and promote G1/S transition 9, 10. IHC overexpression of p16 is routinely used in clinical diagnostics for identification of HPV‐related neoplasms. Ovarian carcinomas are not associated with HPV infections, but alterations promoting G1/S transition are common, e.g. *RB1*, *CCNE1*, *CCND1*, or *MYC* 6.

Ovarian carcinoma is a biologically heterogeneous disease 11 composed of five main histotypes: HGSC, LGSC, clear cell carcinoma (CCC), endometrioid carcinoma (EC), and MC, which should be studied separately 12. Older studies combining all histotypes showed that either overexpression or complete absence of p16 were associated with unfavorable outcomes 13, 14, 15. Recently, histotype‐specific studies also reported that normal heterogeneous p16 expression was significantly associated with longer progression‐free and overall survival (OS) in two series of 334 and 115 women with HGSC 16, 17. Therefore, we hypothesized that heterogeneous p16 expression reflecting the normal G1/S transition status is associated with a favorable outcome in HGSC compared to absent or block expression reflecting abnormalities of the G1/S cell cycle checkpoint complex. The purpose of this study was to validate whether abnormal p16 expression is associated with an unfavorable OS in HGSC, and to explore prognostic associations in other histotypes using tissue microarrays (TMAs) from the Ovarian Tumor Tissue Analysis (OTTA) consortium 18, 19.

## Methods

### Immunohistochemistry

The study investigators obtained tissue from 7492 patients with a diagnosis of primary ovarian, fallopian tube, or peritoneal carcinoma from 24 study sites (Supporting Information, Table S1). Most of these patients also participated in previous OTTA studies 18, 19, 20, and all studies received ethics board approval for tumor profiling. TMAs were constructed containing 1–6 cores of 0.6–1.0 mm in diameter from formalin‐fixed paraffin embedded tissue representing tumor from each patient. p16 IHC was performed centrally at two institutions: Genetic Pathology Evaluation Centre, University of British Columbia, and Calgary Laboratory Services, University of Calgary, Canada. TMAs were stained in five batches with three different protocols (Table S2) using the same antibody (clone E6H4, CINtec, mtm laboratories). Three staining patterns were recorded: absent, heterogeneous and block (Figure S1). Block expression was distinguished from heterogeneous staining by using the recommendation for p16 interpretation from LAST 4; that is, block expression is characterized by diffuse staining of tumor cells in nuclear and/or cytoplasmic compartment with at least moderate intensity with virtually no negative tumor cell clusters. Interobserver reproducibility between two observers (PR and MK) was assessed for a subset of 120 cases. Seventeen studies were scored by PR and the remainder by MK. Cases represented by more than one core and discordant cores were consolidated as heterogeneous if any of a given case score was heterogeneous.

### *CDKN2A* mRNA analysis

A subset of 2280 cases had *CDKN2A* mRNA expression data from NanoString n‐counter analysis. RNA was extracted from 10 μm sections from formalin fixed paraffin embedded (FFPE) tissue blocks, which were macrodissected to avoid adjacent benign tissue but included tumor stroma using the Qiagen miRNeasy (Qiagen Inc. Toronto, Ontario, Canada) FFPE protocol and quantitated on a Nanodrop spectrophotometer (Thermo‐Fisher Scientific, Waltham, MA, USA). After mixing 500 ng of total RNA per sample with a custom codeset (NanoString Technologies Inc, Seattle, WA, USA) and hybridization buffer (NanoString), hybridization was performed using a Tetrad 2 thermal cycler (Bio Rad Laboratories Inc, Hercules, CA, USA) for 16 or 20 h and then analyzed on a nCounter Digital Analyzer (NanoString). Data was normalized to housekeeping genes (*RPL19, ACTB, PGK1, SDHA,* and *POLR1B*) and pre‐processed to a reference of 3 pooled ovarian cancer specimens as described previously 21. We interrogated the cBioportal for associations of *CDKN2A* alterations with OS in HGSC from TCGA 22, 23.

### Statistical tests

Morphology‐based histotype was derived from pathology reports with or without review of reports or slides (Table S1). Because some HGSC were mistakenly classified as other histotypes in the past, we used the highly specific WT1(+)/p53(mutant) IHC combination to reclassify those to HGSC 24. We excluded 409 cases owing to diagnosis other than the five major histotypes, 393 cases being uninterpretable, 31 cases with a combination of absence and block staining, and 134 cases with missing clinical follow‐up data. The final sample size was 6525 (Table 1). The median time from diagnosis to enrollment was 0 days (interquartile range 0–182 days). Patients (*n* = 331) with missing data for either age or time from diagnosis to enrolment were not part of the multivariate survival analysis.

**Table 1:** Clinical characteristics

	High‐grade serous carcinoma	Low‐grade serous carcinoma	Clear cell carcinoma	Endometrioid carcinoma	Mucinous carcinoma
Number of cases, *n* (%)	4334 (66.4)	205 (3.1)	717 (11.0)	882 (13.5)	387 (5.9)
Age at diagnosis, years, mean ± SD	59.7 ± 10.7	53.8 ± 12.7	56.0 ± 11.4	54.8 ± 12.0	54.5 ± 14.8
Stage, *n* (%)					
I/II	822 (19.5)	62 (32.3)	550 (78.2)	703 (83.5)	283 (81.9)
III/IV	3402 (80.5)	130 (67.7)	154 (21.8)	139 (16.5)	67 (19.1)
Unknown	110	13	13	40	37
Macroscopic residual disease, *n* (%)					
Absent	1028 (43.7)	64 (49.6)	349 (81.0)	393 (88.3)	163 (77.2)
Present	1323 (54.3)	65 (50.4)	82 (19.0)	52 (11.7)	51 (23.8)
Unknown	1983	76	286	437	173
Outcome					
Five year survival, % ± SE^1^	40.7 ± 0.8	61.9 ±3.7	63.4 ± 1.9	81.0 ± 1.5	65.3 ± 2.7
Total months followed for censored patients, months, mean ± SD^1^	87 ± 41	80 ± 43	104 ± 39	101 ± 39	97 ± 41
p16 expression, *n* (%)					
Heterogeneous	1627 (37.5)	167 (81.5)	471 (65.7)	676 (76.7)	171 (44.2)
Absent	267 (6.2)	25 (12.2)	146 (20.4)	127 (14.4)	194 (50.1)
Block	2440 (56.3)	13 (6.3)	100 (13.9)	79 (8.9)	22 (5.7)

1 Follow‐up is right‐censored at 12 years post‐diagnosis.

Associations of p16 IHC expression and *CDKN2A* mRNA expression with demographic and clinical variables were examined using the chi‐square test and Kruskal–Wallis test, as appropriate. We examined interobserver heterogeneity of p16 interpretation using Kappa coefficients. For individuals with multiple tumor cores, we examined intratumoral heterogeneity of p16 expression using percent discordance. We assessed correlations between (the ordinally scaled) p16 staining values and *CDKN2A* mRNA expression using Pearson correlation coefficients.

The primary end point for survival analysis was death from any cause. We chose right censoring of follow‐up at 12 years post‐diagnosis to mitigate against deaths from noncancer‐related causes. Kaplan–Meier survival curves and corresponding log‐rank tests were generated to visually assess associations of p16 expression with survival. Cox proportional hazards regression was used for multivariable assessment of hazard ratios (HRs). Models were adjusted for the following confounding factors: study site, age (continuous), FIGO stage (categorized into I/II, III/IV, and missing variable), and residual disease (categorized as absent, i.e. no residual disease, present, and missing). We used left truncation to account for the enrollment of prevalent cases in some studies. We tested whether histotype modified the association between p16 IHC expression and OS by fitting and testing corresponding interaction terms. We assessed the functional form of the association between *CDKN2A* mRNA expression levels and OS in HGSC using penalized B‐splines 25, adjusting for the same potential confounding variables as described above. All statistical tests were two‐sided, and analyses were carried out using RStudio (Boston, MA, USA) or JMP 13.0.0 (SAS, Cary, NC, USA). This study adhered to the REMARK criteria 26.

## Results

### p16 protein and CDKN2A mRNA expression across histological types

Table 1 shows the characteristics of the study sample. The histotype distribution is similar to population‐based cohorts except for a slightly higher frequency of MC 27. Inter‐observer agreement for interpretation of p16 IHC was excellent (Cohen's kappa of 0.92). Of the 6525 women, 4046 (62.0%) had more than one interpretable tissue core and, of those, 12.9% had a discordant interpretation between cores. This moderate degree of intratumoral heterogeneity was not statistically different between histotypes (*p* = 0.11), ranging from 7.4% for LGSC to 16.3% for CCCs. As expected, the distribution of p16 expression categories was significantly different across histotypes (Table 2, *p* < 0.0001) 28.

**Table 2:** Association of p16 expression and OS by histotype

Histotype	Expression	*N*	HR (95% CI)	*P* value
High‐grade serous	Heterogeneous	1550	ref	0.68
	Absent	244	1.06 (0.90–1.25)	
	Block	2292	1.03 (0.95–1.11)	
Low‐grade serous	Heterogeneous	166	ref	0.001
	Absent	25	2.95 (1.61–5.38)	
	Block	13	1.54 (0.72–3.29)	
Clear cell	Heterogeneous	463	ref	<0.001
	Absent	138	0.67 (0.47–0.96)	
	Block	92	2.02 (1.47–2.77)	
Endometrioid	Heterogeneous	650	ref	0.004
	Absent	117	0.98 (0.66–1.45)	
	Block	73	1.88 (1.30–2.75)	
Mucinous	Heterogeneous	163	ref	0.80
	Absent	187	1.05 (0.72–1.55)	
	Block	21	1.28 (0.61–2.64)	

Adjusted for study, age, time interval, stage and residual tumor; ref, reference.

Smaller sample size is due to availability of age and time interval information.

**Figure 1:**
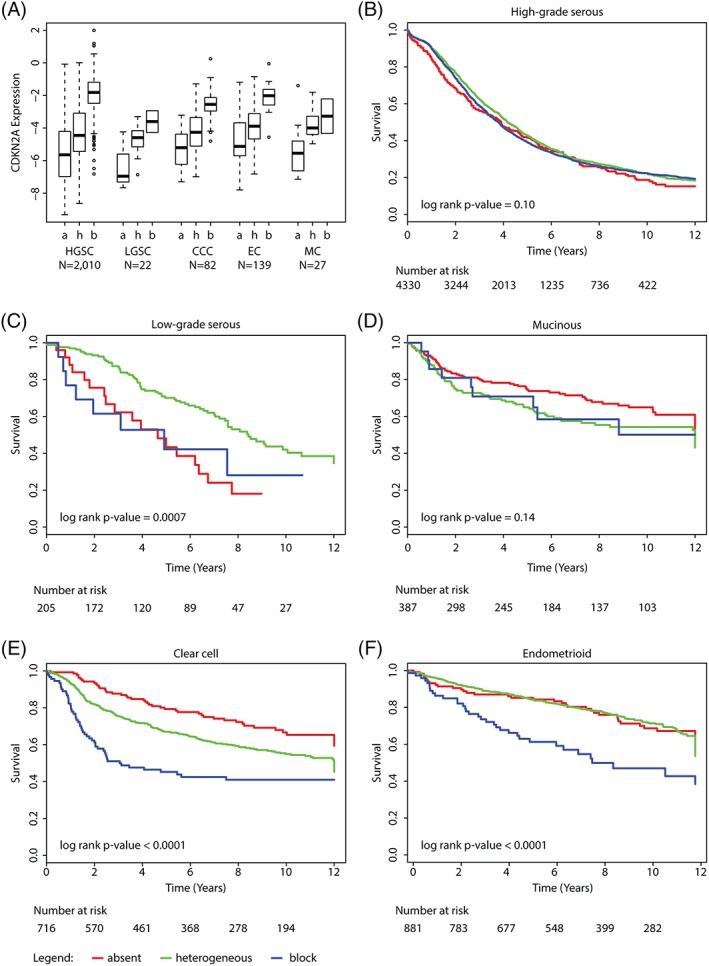
Associations of p16 protein expression with *CDKN2A* mRNA expression and survival by histotype. (A) Comparison of *CDKN2A* mRNA values (y‐axis) with p16 scoring categories (*x*‐axis), by the five major histotypes. a = p16 absence, h = p16 heterogeneous, b = p16 block score, respectively. Kaplan–Meier OS curves of p16 expression within (B) high‐grade serous, (C) low‐grade serous, (D) mucinous, (E) clear cell and (F) endometrioid carcinoma.

A subset of 2280 cases (2010 HGSC, 22 LGSC, 139 EC, 82 CCC, and 27 MC) had corresponding *CDKN2A* mRNA expression data. *CDKN2A* mRNA expression was significantly higher in HGSC [normalized mean −2.98 (95% CI ‐3.06 to −2.90)] compared to LGSC [normalized mean −4.90 (95% CI −5.39 to −3.19)], EC [normalized mean −4.82 (95% CI −5.31 to −4.33)], CCC [normalized mean −4.16 (95% CI −4.51 to −3.81)] and MC [normalized mean −4.50 (95% CI −5.07 to −3.93), for all *p* < 0.0001]. We confirmed the bimodal distribution of *CDKN2A* mRNA expression in HGSC as previously observed in the TCGA data set 22, 23 (Figure S2). *CDKN2A* mRNA expression correlated with p16 IHC scores (*r* = 0.69) overall, and for the specific histotypes (*r* = 0.69 for HGSC, 0.57 for LGSC, 0.62 for CCC, 0.80 for EC and 0.69 for MC, Figure 1A).

### Association of p16 protein and CDKN2A mRNA expression and OS in HGSC and LGSC

For HGSC patients, the Kaplan–Meier survival curve showed no difference in OS for the three p16 immunohistochemical expression patterns (Figure 1B, *p* = 0.32), which was supported by HRs near 1.0 after controlling for the study, age, time to enrollment, stage and residual disease (Table 2).

For mRNA expression in HGSC, we used several different groupings (median split, tertiles) as well as different cut‐offs for dichotomization (mean = −3.03, visual inspection to separate bimodal peaks = −3.7) but there was no association of *CDKN2A* mRNA levels with OS. Alternatively, we examined the functional form of the association between *CDKN2A* expression and OS in HGSC using penalized B splines 25. Analyses revealed relatively flat HRs across the entire spectrum of mRNA values with a 95% confidence band that always included an HR of 1.0 (Figure S3). By interrogating 489 HGSC from TCGA *via* the cBioPortal 22, 23, 92 (19%) showed downregulation of *CDKN2A*, which was also not associated with survival (p = 0.27).

In contrast, for patients with LGSC, the 5‐year survival rate was significantly lower in tumors with absent p16 expression (Figure 1C, 43.4%, SE 10.7%) and in tumors with block p16 expression (42.2%, SE 14.7%) compared to heterogeneous expression (70.1%, SE 3.8%, *p* = 0.0005). This was also significant in multivariate analysis for the absence of p16 (Table 2). The limited number of *CDKN2A* mRNA expression values for non‐HGSC precluded us from examining associations within those histotypes.

### No association of p16 protein expression and OS in MC

Figure 1D shows no differences in 5‐year survival for MC with heterogeneous (63.7%, SE 3.8%), absent (73.3%, SE 3.2%) or block expression (72.2%, SE 9.7%, *p* = 0.12). There was no significant association in multivariate analysis (Table 2).

### Association of p16 protein expression and OS in endometriosis related ovarian carcinomas

For CCC, 5‐year survival was more than 20% lower for women with tumor block expression of p16 (45.2%, SE 5.0%) compared to heterogeneous staining (67.0%, SE 2.2%, *p* < 0.0001, Figure 1E). Similarly, for EC, 5‐year survival was more than 20% lower for women with tumor block expression of p16 (63.0%, SE 5.8%) compared to heterogeneous staining (85.0%, SE 1.4%, *p* < 0.0001, Figure 1F) or absent staining (85.2%, SE 3.3). In multivariate analysis, there was a significantly increased risk of death for patients with CCC or EC block staining, with HRs of 2.02 (95% CI: 1.47–2.77) and 1.88 (95% CI: 1.30–2.75), respectively (Table 2).

### Pooled association of p16 protein expression and OS, and tests of effect modification by histotype

In analyses that combined all five major histological subtypes, women whose tumors exhibited with block expression had poorer OS (HR 1.13, 95% CI 1.06–1.22) than those with absent or heterogeneous expression (Figure S4). The associations between p16 expression and survival were strikingly different across histotypes (Cox regression test for interaction *p* = 1.4 × 10^−8^).

### Association of p16 protein expression with clinicopathological parameters within histotypes

In EC, a greater proportion of cases with p16 block expression were diagnosed at stage III/IV (33.8% compared to 14.5% for heterogeneous staining, *p* < 0.0001) and grade 3 (45.7% compared to 16.0% for heterogeneous staining, *p* < 0.0001, Table S3). CCC cases with p16 block expression were more likely to have residual disease at initial surgery (34.6% compared to 17.1% for heterogeneous staining, *p* = 0.0067). In LGSC, p16 expression was not associated with age, stage or presence of residual tumor. Associations for HGSC and MC are shown in Table S3. Notably, there was no association for p16 expression status with *BRCA1* or *BRCA2* mutation status for the subset of HGSCs with available mutation data (*n* = 1370, *p* = 0.43).

## Discussion

Our investigation showed that associations of p16 staining pattern with OS differ across ovarian carcinoma histotypes. Block p16 expression was significantly associated with shorter survival for endometriosis‐related ovarian carcinomas. In contrast, absence of p16 expression predicted shorter survival in LGSC while no survival associations are observed for MC and HGSC.

In contrast to previous studies 16, 17, we provide strong evidence against a clinically or biologically relevant survival association of p16 expression in HGSC. Our large sample size greatly reduces the potential of this lack of association being a false negative finding. Using our observed sample size and a two‐sided test of hypothesis with Type I error rate of 0.05, we would have 80% power to detect a HR as low as 1.21 comparing absence and 1.10 comparing block to heterogeneous expression, respectively. This null conclusion is supported by a lack of association between OS and *CDKN2A* mRNA expression data, which correlated positively with p16 IHC‐based protein expression. In keeping with the recommendations from the Institute of Medicine for validation of biomarker studies 29, we used the same antibody and the same scoring system as previous studies. We found excellent interobserver reproducibility regarding the IHC interpretation and similar frequencies of the three staining patterns in HGSC compared to previous studies; and it is, therefore, unlikely that technical or interpretational differences can explain differences in our results from those published previously. We think that the large sample size used in the current study compared to prior studies of HGSC avoided a false positive finding 16, 17. Since p16 block staining is a surrogate for various retinoblastoma pathway alterations, we speculate that different underlying alteration might explain the lack of outcome associations for p16. For example, prognostically opposing underlying alterations (e.g. favorable pRB loss *versus* unfavorable *CCNE1* amplifications), which result in the same p16 block staining, may neutralize each other 30, 31.

Exploring other histotypes, we demonstrate for the first time that block p16 expression is significantly associated with OS in both endometriosis‐associated histotypes: EC and CCC. Overall, those histotype‐specific differences would not have been revealed in a combined histotype analysis and corroborate that biomarker analyses should be done stratified by histotype 12. Yet the subsets of p16 block expression that were significantly associated with unfavorable prognosis were small: 9 and 14% of EC and CCC, respectively. EC is usually associated with a favorable outcome and some patients do not require chemotherapy or could be considered for hormonal therapy if hormone receptors (ER, PGR) are expressed 18, 32. However, estrogen receptor positive Luminal B breast cancers with loss of pRB function and high p16 expression are unresponsive to hormonal therapy 33. Our data suggest that further study of p16 as part of a biomarker panel that identifies EC with unfavorable prognosis would help triage patients to earlier aggressive therapy in the low stage setting, which may not be amenable to hormonal therapy.

We observed a similar negative association between block p16 expression and OS for CCC. Women diagnosed with CCC have a relatively unfavorable prognosis, in part because these tumors are chemotherapy‐resistant and alternative therapeutic options are sparse 34. Among those, radiation has been suggested for CCC 35 and perhaps p16 expression can be assessed for prediction of response to radiation as suggested from other cancer sites 3, 36. The survival associations specifically observed for p16 block staining in the two endometrioisis‐associated histotypes somewhat serves as a cross‐validation. Yet it does not preclude differences in the underlying mechanisms, e.g. *CCNE1* copy number gain and overexpression have been reported for CCC but not EC 37. As a limitation, we observed a moderate degree of intratumoral heterogeneity, which was highest in CCC, using TMAs in size akin to pretreatment omental core biopsies. However, CCCs are usually treated by upfront surgery and the p16 assessment on a full histological section should mitigate against intratumoral heterogeneity.

We also observed a significant association with OS in patients with LGSC. In contrast to the block staining in endometriosis‐associated carcinomas, complete absence was associated with unfavorable outcome in LGSC in keeping with the tumor suppressor function. Although investigating the underlying mechanism of absence of p16 expression is beyond the scope of the current study, the 12% of LGSC showing complete absence of p16 by IHC is strikingly similar to the 15% frequency of the homozygous deletion of the *CDKN2A* locus reported by Hunter *et al* 7. We have previously shown that progesterone receptor (PGR) expression is a favorable prognostic factor in LGSC 18. Perhaps PGR and p16 status could help to stratify LGSC regarding prognosis 38. Another consideration is the possible predictive utility of absent p16 expression for CDK4 inhibitors as suggested in clinical trials for breast and other cancers 39. Konecny *et al* demonstrated that low p16 expression in pRB‐proficient tumors was correlated with *in vitro* response to CDK4 inhibitors 40. Since other treatment options are limited for LGSC, this may represent an interesting option for recurrent LGSC, a disease often affecting younger women. There was a non‐significant trend for the few LGSC with p16 block expression to have an unfavorable outcome but CKD4 inhibitors will be ineffective in tumors with p16 block expression because p16 block expression already indicates futile intrinsic CDK4 inhibition.

There was no significant prognostic association of p16 expression within MCs despite their having the highest frequency of complete absence (50%) across histotypes. This frequency is slightly higher than the 39% (*n* = 12/31) frequency of the combined corresponding molecular alterations (homozygous *CDKN2A* deletion or loss of functional mutations) reported by Ryland *et al* 8. Absence of p16 expression was most frequently observed in low stage MC and we speculate that a portion of those may be from low transcriptional activity.

A strength of this study is the large sample size providing excellent power to examine differences in protein expression patterns for the most common histotype (HGSC) as well as reasonable power to discern differences within EC and CCC. The lower sample sizes for MC and LGSC reduced our ability to detect differences. We used a diagnostic biomarker panel of WT1/p53 to limit the number of misclassified HGSC into other histotype categories. Within the OTTA consortium, we had the opportunity to analyze protein and mRNA data. Survival analyses were adjusted for confounding factors such as age, stage and residual disease. As a limitation, some of the study sites did not collect detailed treatment data; as such adjustment for treatment was not feasible. However, cases were collected throughout an era of relatively homogeneous standard adjuvant therapy consisting of platinum‐taxane chemotherapy before the introduction of neoadjuvant chemotherapy or PARP inhibitors.

This large‐scale collaborative study did not validate p16 expression as a prognostic marker in HGSC. The frequent block expression as a surrogate for abnormal retinoblastoma pathway activation warrants further study of individual pathway members. The intriguing prognostic associations in endometriosis‐associated EC and CCC make p16 a promising prognostic biomarker that requires further independent validation. The absence of p16 in a subset of LGSC calls for independent validation as a prognostic marker as well as investigation as a predictive marker for CDK4 inhibitors.

## Author contributions statement

DGH and MK conceived the study design. PFR, CC, MSA, SMA, CW, AT and MK carried out experiments and interpreted results. RAV and MK analyzed the data. All authors collected data. PFR, RAV and MK wrote the first draft. All authors were involved in writing the paper and had final approval of the submitted and published versions.

## Supporting information


**SUPPLEMENTARY MATERIAL ONLINE**

**Figure S1.** p16 immunohistochemistry. (A) Heterogeneous staining showing variable staining in tumor cells. (B) Complete absence in tumor with some normal cells staining. (C) Block staining with cytoplasmic and nuclear p16 expression in all tumor cells

**Figure S2.** Normalized *CDKN2A* mRNA expression values for HGSC showing bimodal distribution

**Figure S3.** Assessment of the Functional Form of *CDKN2A* mRNA values with overall survival in a subset of 1882 women with HGSOC. Vertical dotted lines indicate the mean values of mRNA expression for tumors with absence of p16 staining (left‐most line), heterogeneous staining (middle line) and block staining (right‐most line)

**Figure S4.** Kaplan–Meier overall survival curves of p16 expression in pooled analysis combining all histotypes

**Table S1.** Participating studies 41, 42, 43, 44, 45, 46, 47, 48, 49, 50, 51, 52, 53, 54, 55, 56, 57, 58, 59, 60, 61, 62, 63

**Table S2.** Immunohistochemical staining protocols

**Table S3.** Association of p16 expression with clinicopathological parameters
