# Supplementary figures and images for: Association of p16 expression with prognosis varies across ovarian carcinoma histotypes: an Ovarian Tumor Tissue Analysis consortium study
Source: J Pathol Clin Res. 2018 Sep 21;4(4):250–61. doi: 10.1002/cjp2.109 (PMC6174617; doi:10.1002/cjp2.109)

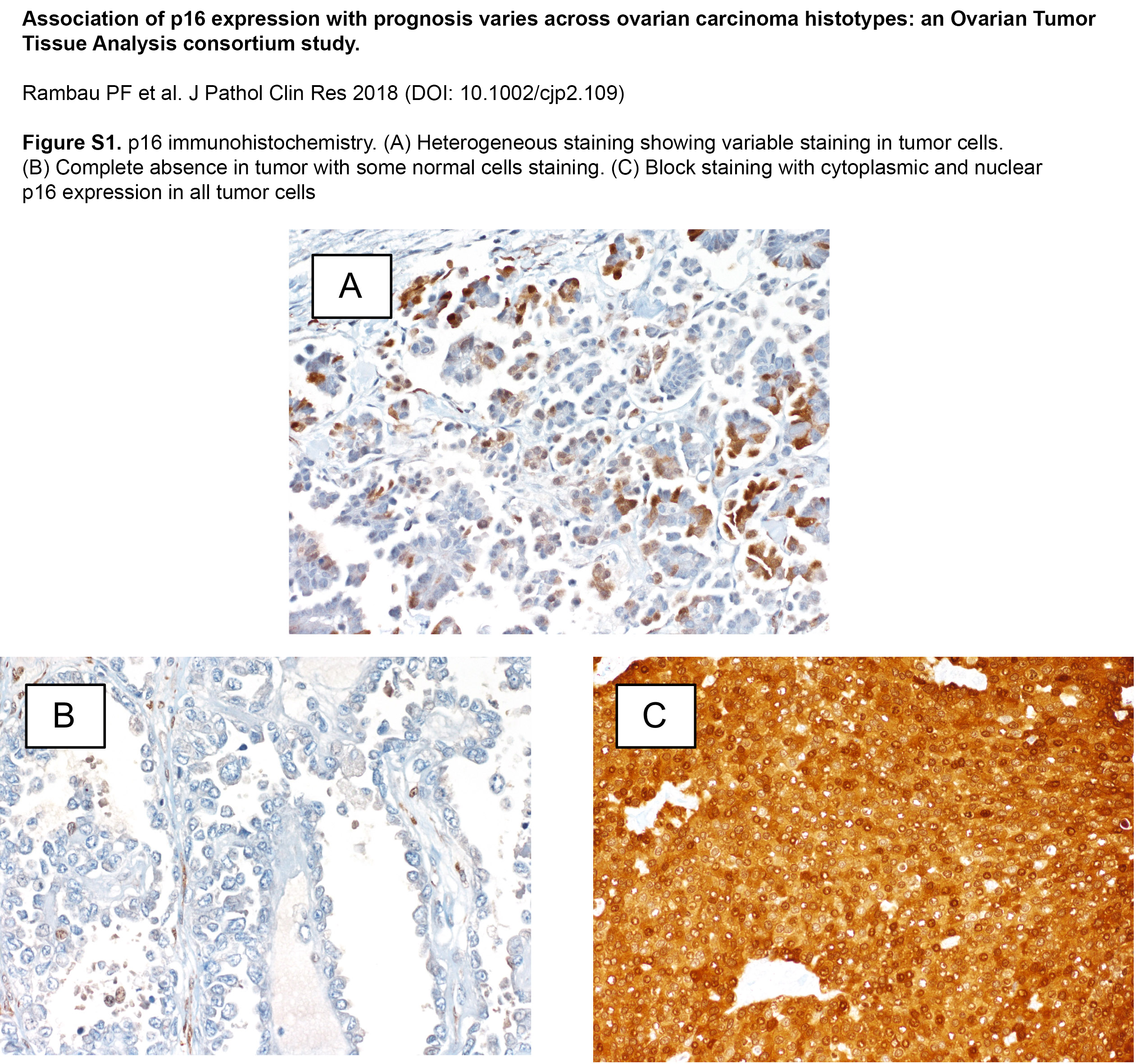

Supplement: Supplementary file 1 — Figure S1. p16 immunohistochemistry. (A) Heterogeneous staining showing variable staining in tumor cells. (B) Complete absence in tumor with some normal cells staining. (C) Block staining with cytoplasmic and nuclear p16 expression in all tumor cells [file CJP2-4-250-s001.tif]

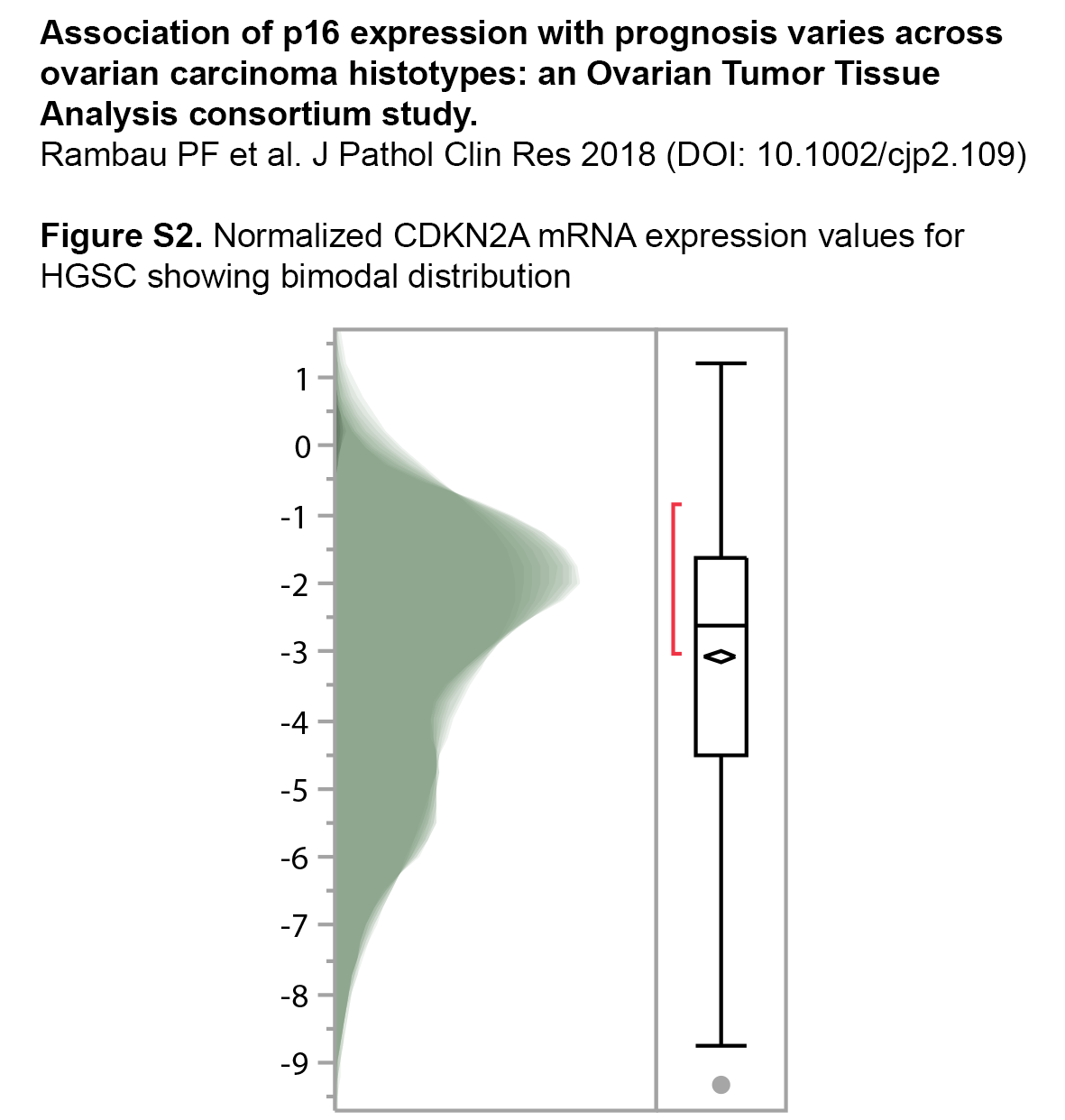

Supplement: Supplementary file 2 — Figure S2. Normalized CDKN2A mRNA expression values for HGSC showing bimodal distribution [file CJP2-4-250-s003.tif]

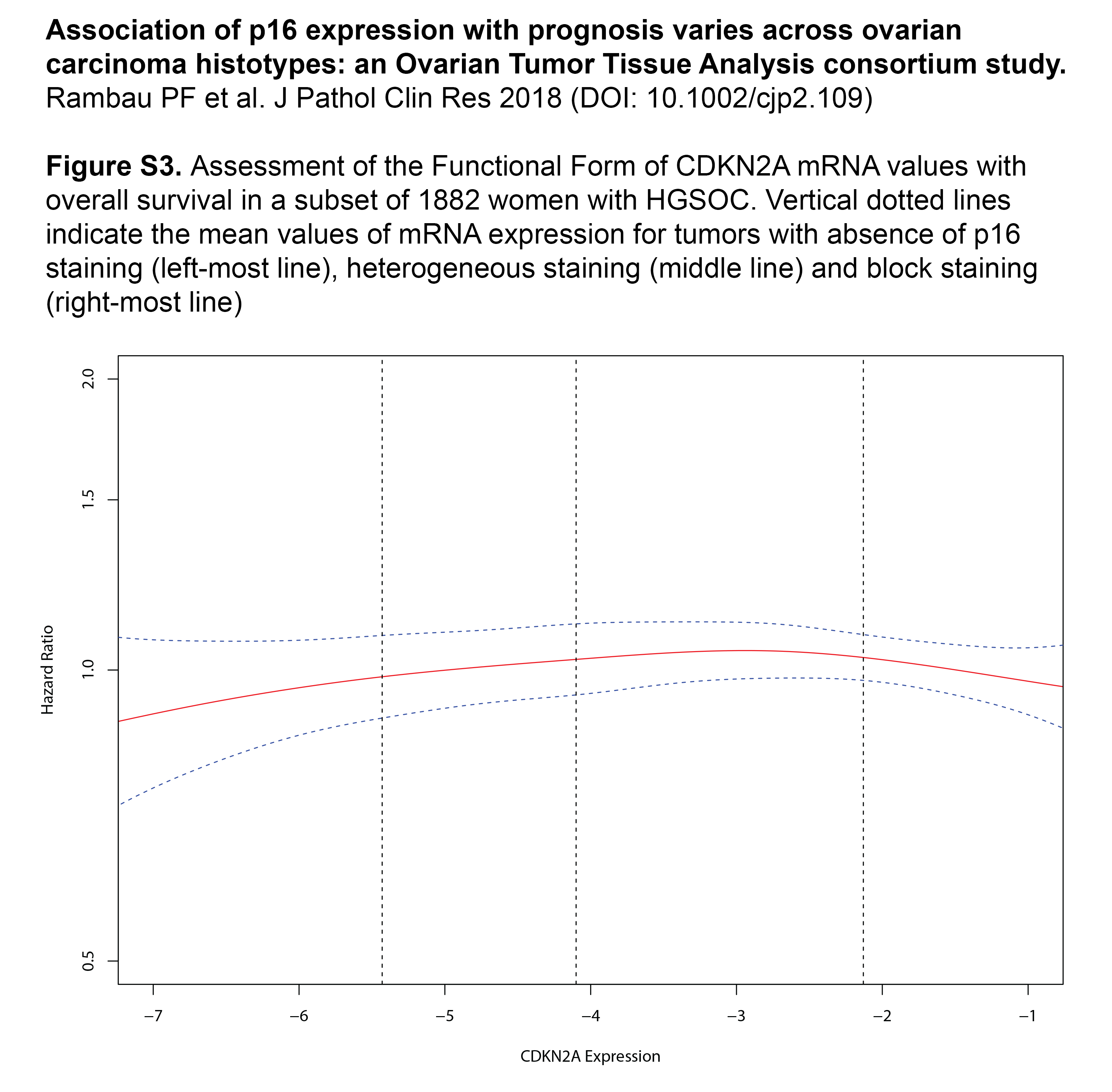

Supplement: Supplementary file 3 — Figure S3. Assessment of the Functional Form of CDKN2A mRNA values with overall survival in a subset of 1882 women with HGSOC. Vertical dotted lines indicate the mean values of mRNA expression for tumors with absence of p16 staining (left‐most line), heterogeneous staining (middle line) and block staining (right‐most line) [file CJP2-4-250-s004.tif]

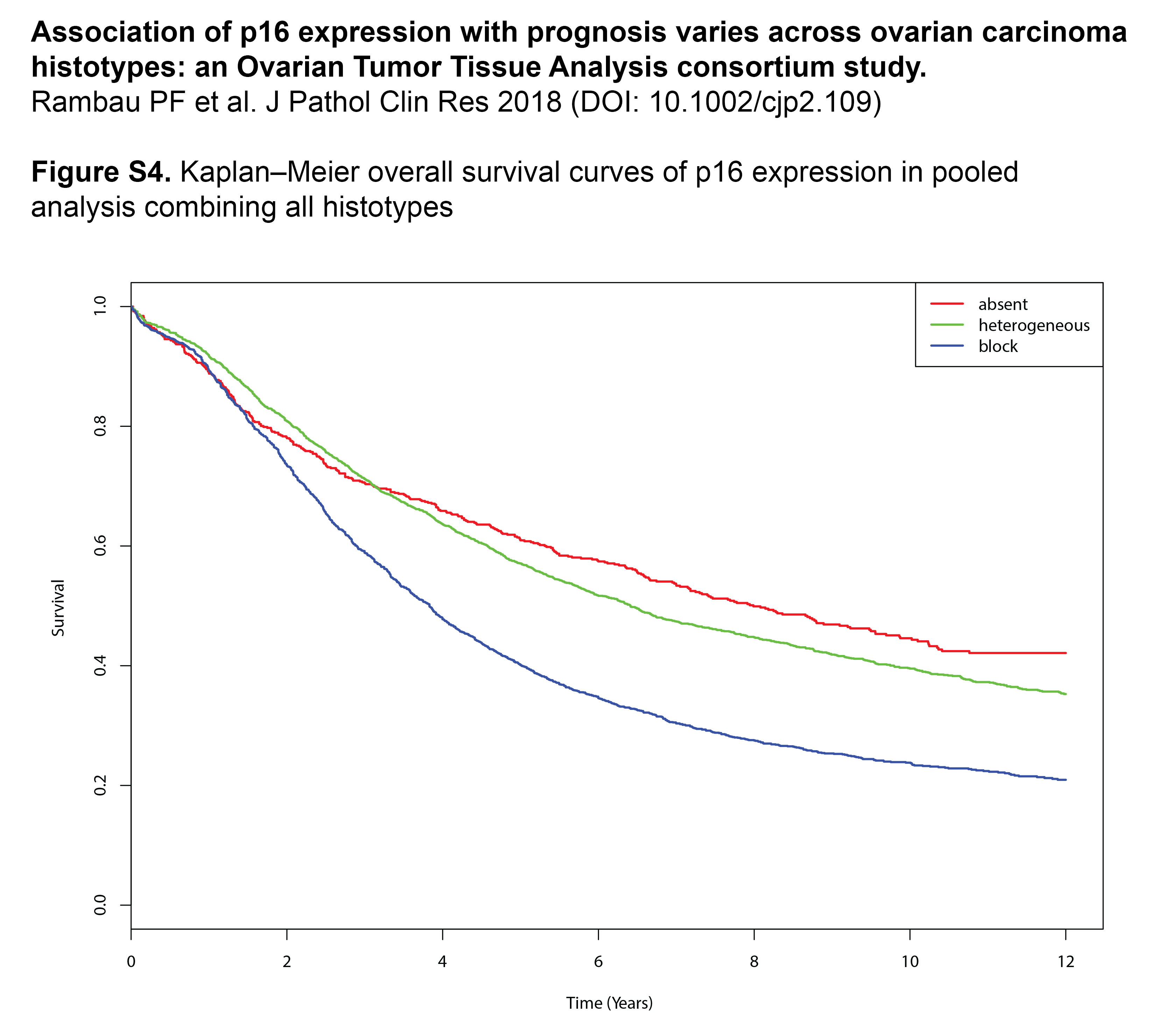

Supplement: Supplementary file 4 — Figure S4. Kaplan–Meier overall survival curves of p16 expression in pooled analysis combining all histotypes [file CJP2-4-250-s002.tif]
