# Supplementary material for: Association of p16 expression with prognosis varies across ovarian carcinoma histotypes: an Ovarian Tumor Tissue Analysis consortium study
Source: J Pathol Clin Res. 2018 Sep 21;4(4):250–61. doi: 10.1002/cjp2.109 (PMC6174617; doi:10.1002/cjp2.109)
Supplement: Supplementary file 5 — Table S1. Participating studies 41, 42, 43, 44, 45, 46, 47, 48, 49, 50, 51, 52, 53, 54, 55, 56, 57, 58, 59, 60, 61, 62, 63 [file CJP2-4-250-s005.docx]

**Association of p16 expression with prognosis varies across ovarian carcinoma histotypes: an Ovarian Tumor Tissue Analysis consortium study.**

Rambau PF et al. J Pathol Clin Res 2018 (DOI: 10.1002/cjp2.109)

**Table S1. Participating studies**

| **Study** | **Name** | **Ref-erence** | **Location** | **Years** | **Ascertainment of Patients and Clinical Data** | **Pathology Data and Review** | **N (%)** | **High-grade serous N (%)** |  |  |
| --- | --- | --- | --- | --- | --- | --- | --- | --- | --- | --- |
| AOC | Australian Ovarian Cancer Study | (4[1](#_ENREF_1)) | Australia | 2002-2006 | Treatment centers throughout Australia; cancer registries serving Queensland, South and West Australia; regular follow-up visits | Pathology reports and diagnostic slides reviewed by panel of gynecologic pathologists | 335 (5.1) | 321 (7.4) |  |  |
| AOV | Alberta Ovarian Tumor Types Study | (4[2](#_ENREF_2)) | Canada | 1978-2010 | Population-based Alberta Cancer Registry; annual updates are performed for vital statistics | Pathology reports and histological slides review by the study pathologist | 460 (7.0 | 76 (1.7) |  |  |
| BAV | Bavarian Ovarian Cancer Study | (4[3](#_ENREF_3)) | Germany | 2002-2006 | Gynecologic Oncology Center at the Comprehensive Cancer Center Erlangen-Nuremberg | Centralized review of pathology reports and histological slides for all patients by study pathologists | 219 (3.4) | 147 (3.4) |  |  |
| BGS | Breaktfhrough Generations Study | - | UK | 1988-2013 | Follow-up of a national general population cohort study | Pathology reports | 60 (0.9) | 38 (0.9) |  |  |
| BRZ | Ribeirao Preto Ovarian Cancer Study | - | Brazil | 1987-2010 | University Hospital of Ribeirao Preto School of Medicine (HCRP), case series with prospective follow up | Pathology reports and histologic slides reviewed by HCRP gynecologic pathologists | 104 (1.6) | 62 (1.4) |  |  |
| CAL | Calgary Serous Carcinoma Study | ([4](#_ENREF_4)4) | Canada | 2003-2007 | Hospital based retrospective observational study | Histological review of all slides by study pathologist supported by centralized biomarker analysis | 105 (1.6) | 78 (1.8) |  |  |
| CNI | CNIO Ovarian Cancer Study | (4[5](#_ENREF_5)) | Spain | 2006-2013 | Hospitals in Madrid in Medical Oncology Divisions | Pathology information was obtained from medical charts of the patients used in the Medical Oncology Units | 121 (1.8) | 62 (1.4) |  |  |
| DOV | Diseases of the Ovary and their Evaluation | - | US | 2002 to 2009 | Fred Hutchinson Cancer Research Center | Two stage review: H&E stained slides centrally reviewed by the expert study pathologist who assigned ICD-O codes for morphology and tumor grade. Standardized Pathologic Review. As a second means of assigning histotype, expert pathologists in Vancouver, British Columbia re-reviewed the H&E stained slide of the primary tumor and assigned histotype using the 2014 WHO diagnostic classification guidelines | 999 (15.3) | 749 (17.3) |  |  |
| DUK | Duke University Medical Center | - | US | 2008-2009 | Duke University Medical Center, North Carolina | H&E stained slides reviewed by expert study pathologist | 43 (0.7) | 43 (0.6) |  |  |
| GER | Germany Ovarian Cancer Study | (4[6](#_ENREF_6)) | Germany | 1993-1996 | 26 hospitals in the study regions | Pathology reports were requested from the respective pathology institutes. Tissue samples were provided by the tissue bank of the National Center for Tumor Diseases (NCT, Heidelberg, Germany) in accordance with the regulations of the tissue bank and the approval of the ethics committee of Heidelberg University and by other pathology institutes. Histological slides were reviewed by gynecologic pathologist at the University of Heidelberg | 84 (1.3) | 61 (1.4) |  |  |
| HAW | Hawaii Ovarian Cancer Study | (4[7](#_ENREF_7), 4[8](#_ENREF_8)) | US | 1993-2008 | Hawaii Tumor Registry and medical records | Pathology reports and histological slides reviewed by study pathologist | 119 (1.8) | 65 (1.5) | |  |
| HOP | Hormones and Ovarian Cancer PrEdiction | (4[9](#_ENREF_9)) | US | 2003-2009 | Hospital registries and active surveillance of medical practices in Western PA, Northeastern OH, and Western NY | Medical chart review for all cases | 38 (0.6) | 28 (0.7) | |  |
| LAX | Women's Cancer Research Program - Cedars-Sinai Medical Center | ([50](#_ENREF_10)) | US | 1989-present | Women's Cancer Program Biorepository | Pathology reports and histological slides reviewed by the Department of Pathology and Laboratory Medicine at Cedars-Sinai Medical Center | 246 (3.8) | 245(5.6) | |  |
| MAL | Malignant Ovarian Cancer Study | ([51](#_ENREF_11), [52](#_ENREF_12)) | Denmark | 1994-1999 | Gynecological departments in Copenhagen, Frederiksberg and 7 surrounding counties | Review of pathology reports for all patients and histological slides for 30% by gynecologic pathologist | 64 (1.0) | 7(0.1) | |  |
| MAY | Mayo Clinic Ovarian Cancer Study | ([53](#_ENREF_13)) | US | 2000-2013 | Mayo Clinic medical records and death certificates | Pathology reports and histologic slides reviewed by Mayo Clinic gynecologic pathologists | 675 (10.3) | 524 (12.1) | |  |
| NOT | Nottingham Study | ([54](#_ENREF_14)) | UK | 1991-2011 | Hospital records and Trent cancer registry | Pathology reports reviewed by gynecologic pathologist | 438 (6.7) | 289 (6.7) | |  |
| POC | Polish Ovarian Cancer Study | ([55](#_ENREF_15)) | Poland | 2000-2003 | Hospital records and cancer registries serving Warsaw and Lodz | Histological slides reviewed by study pathologist | 126 (1.9) | 90 (2.0) | |  |
| SEA | Study of Epidemiology and Risk Factors in Cancer Heredity | ([56](#_ENREF_16)) | UK | 1998-present | Eastern Region Cancer Intelligence Unit, West Midlands Cancer Intelligence Unit, and multiple cancer networks | Pathology reports and histological slides reviewed by study pathologist | 504 (7.7) | 297 (6.8) | |  |
| STA | Genetic Epidemiology of Ovarian Cancer Study | (57) | US | 1997-2001 | Greater Bay Area Cancer Registry | Pathology reports and histological slides reviewed by study pathologist | 302 (4.6) | 207 (4.8) | |  |
| TUE | Tuebingen University Hospital | (58) | Germany | 1999-2008 | Department of Obstetrics and Gynaecology, Eberhard Karls Universitats Tübingen, Tübingen Germany | Pathology reports and histologic slides reviewed by gynecologic pathologist | 194 (3.0) | 155 (3.6) | |  |
| TVA | Ovarian Cancer in Alberta | (59) | Canada | 2004-2012 | Alberta Cancer Registry and Provincial Cancer Treatment Centers | Pathology reports and histologic slides reviewed by gynecologic pathologist | 145 (2.2) | 90 (2.1) | |  |
| UKO | United Kingdom Ovarian Cancer Population study | ([60](#_ENREF_19)) | UK | 2006-2010 | Ten major Gynecologic Oncology NHS centers in England, Wales and Northern Ireland; cancer registries; NHS Information Centre for Health and Social Care (England and Wales) and Central Services Agency (Northern Ireland) | Central review of pathology reports by gynecologic oncologist | 101 (1.6) | 72 (1.7) | |  |
| VAN | Vancouver Ovarian Cancer Study | (61, [62](#_ENREF_21)) | Canada | 1984-2000 | Ovarian Cancer Registry serving British Columbia and the Cheryl Brown Outcomes Unit | Central review of pathology reports and histological slides by University of British Columbia pathologists | 801 (12.2) | 443 (10.2) | |  |
| WMH | Westmead Hospital, Gynaecological Oncology Biobank (GynBiobank) | ([63](#_ENREF_22)) | Australia | 1992-present | The Crown Princess Mary Cancer Centre and affiliated hospitals | Pathology reports and diagnostic slides reviewed by panel of gynecologic pathologists | 242 (3.7) | 185 (2.8) | |  |
|  | **TOTAL** |  |  |  |  |  | **6525** | **4334** | | |

*****See reference list on supplementary references
