# Supplementary material for: Association of p16 expression with prognosis varies across ovarian carcinoma histotypes: an Ovarian Tumor Tissue Analysis consortium study
Source: J Pathol Clin Res. 2018 Sep 21;4(4):250–61. doi: 10.1002/cjp2.109 (PMC6174617; doi:10.1002/cjp2.109)
Supplement: Supplementary file 6 — Table S2. Immunohistochemical staining protocols [file CJP2-4-250-s007.docx]

**Association of p16 expression with prognosis varies across ovarian carcinoma histotypes: an Ovarian Tumor Tissue Analysis consortium study.**

Rambau PF et al. J Pathol Clin Res 2018 (DOI: 10.1002/cjp2.109)

**Table S2.** Immunohistochemical staining protocols

| **Platform** | **Cases N (%)** | **Pretreatment** | **Antibody clone/supplier** | **Dilution** | **Detection kit** |
| --- | --- | --- | --- | --- | --- |
| V1-3 GPEC Ventana | 6078 (93%) | Cell Conditioning 1 (CC1) | E6H4(CINtec)/mtm laboratories | Pre-dilute | UltraMap DAB anti-Ms Detection Kit |
| V4 Leica Bond III Calgary | 349 (5%) | Bond Epitope Retrieval Solution 2 (ER2-20) | E6H4(CINtec)/mtm laboratories | 1:24 | Bond polymer refine |
| V5 Dako Omnis, Calgary | 98 (2%) | HIER, pH9 | E6H4(CINtec)/mtm laboratories | 1:24 | DAKO ENVISION FLEX+ |

Controls: High expressor control: high-grade squamous intraepithelial lesion (HSIL); low expressor control: tronsilar squamous epithelium showing scattered epithelial with moderated to strong staining; negative control: normal cervical squamous mucosa showing no staining.

Inter-assay comparison:

N=240 cases were stained with both V3 GPEC and V4 Leica Bond III: kappa =0.83, concordance 91%.

N=151 cases were stained with both V4 Leica Bond III and v5 Dako Omnis, kappa 0.83, concordance 91%
