# Supplementary material for: Association of p16 expression with prognosis varies across ovarian carcinoma histotypes: an Ovarian Tumor Tissue Analysis consortium study
Source: J Pathol Clin Res. 2018 Sep 21;4(4):250–61. doi: 10.1002/cjp2.109 (PMC6174617; doi:10.1002/cjp2.109)
Supplement: Supplementary file 7 — Table S3. Association of p16 expression with clinicopathological parameters [file CJP2-4-250-s006.docx]

**Association of p16 expression with prognosis varies across ovarian carcinoma histotypes: an Ovarian Tumor Tissue Analysis consortium study.**

Rambau PF et al. J Pathol Clin Res 2018 (DOI: 10.1002/cjp2.109)

**Table S3. Association of p16 expression with clinicopathological parameters**

|  | **p16 absent** | **p16 heterogeneous** | **p16 block** | **p-value** |
| --- | --- | --- | --- | --- |
| **High-grade serous** |  |  |  |  |
| Age, mean | 60.4 | 59.1 | 60.0 | 0.015 |
| Pretreatment CA125, mean | 1360 | 2012 | 1286 | 0.055 |
| Stage III,IV (%) | 78.7 | 80.3 | 80.9 | 0.65 |
| Residual tumor present (%) | 64.6 | 52.9 | 57.8 | 0.012 |
| BRCA1/2 mutant (%) | 21.3 | 25.0 | 22.0 | 0.43 |
| **Low-grade serous** |  |  |  |  |
| Age, mean | 52.8 | 53.7 | 55.8 | 0.78 |
| Pretreatment CA125, mean | 442 | 290 | NA | 0.71 |
| Stage III,IV (%) | 58.3 | 68.6 | 75.0 | 0.52 |
| Residual tumor present (%) | 41.2 | 49.5 | 77.8 | 0.19 |
| **Endometrioid** |  |  |  |  |
| Age, mean | 55.7 | 54.5 | 55.3 | 0.56 |
| Pretreatment CA125, mean | 377 | 592 | 677 | 0.69 |
| Stage III,IV (%) | 16.8 | 14.5 | 33.8 | <0.0001 |
| Grade 3 (%) | 21.2 | 16.0 | 45.7 | <0.0001 |
| Residual tumor present (%) | 4.6 | 11.8 | 17.4 | 0.17 |
| **Clear cell** |  |  |  |  |
| Age, mean | 56.9 | 56.1 | 54.4 | 0.24 |
| Pretreatment CA125, mean | 130 | 417 | 239 | 0.71 |
| Stage III,IV (%) | 20.8 | 20.6 | 29.3 | 0.15 |
| Residual tumor present (%) | 15.0 | 17.1 | 34.6 | 0.0067 |
| **Mucinous** |  |  |  |  |
| Age, mean | 53.4 | 56.3 | 50.3 | 0.067 |
| Pretreatment CA125, mean | 97 | 152 | 163 | 0.23 |
| Stage III,IV (%) | 15.6 | 27.6 | 15.8 | 0.028 |
| Residual tumor present (%) | 14.3 | 24.8 | 20.0 | 0.055 |

P-value: Anova for continuous and Pearson Chi square for categorical variables
